# *CsUGT89A2* enhances tea plant resistance to *Toxoptera aurantia* by mediating flavonoid glycosides biosynthesis

**DOI:** 10.1093/hr/uhaf212

**Published:** 2025-08-11

**Authors:** Xingrong Zhou, Dingli Chen, Fengyun Tian, Jie Ma, Mei Chen, Youshudi Xie, Changli Yang, Yanglin Liang, Houhong Xiao, Xue Dong, Di Yang, Yingqin He, Xinlong Dai, Yan Li

**Affiliations:** College of Tea Science, Guizhou University, Guiyang 550025, Guizhou Province, China; College of Tea Science, Guizhou University, Guiyang 550025, Guizhou Province, China; College of Tea Science, Guizhou University, Guiyang 550025, Guizhou Province, China; College of Tea Science, Guizhou University, Guiyang 550025, Guizhou Province, China; College of Tea Science, Guizhou University, Guiyang 550025, Guizhou Province, China; College of Tea Science, Guizhou University, Guiyang 550025, Guizhou Province, China; College of Tea Science, Guizhou University, Guiyang 550025, Guizhou Province, China; College of Tea Science, Guizhou University, Guiyang 550025, Guizhou Province, China; College of Tea Science, Guizhou University, Guiyang 550025, Guizhou Province, China; College of Tea Science, Guizhou University, Guiyang 550025, Guizhou Province, China; School of Pharmaceutical Sciences, Guizhou University, Guiyang 550025, China; College of Tea Science, Guizhou University, Guiyang 550025, Guizhou Province, China; College of Tea Science, Guizhou University, Guiyang 550025, Guizhou Province, China; Guizhou Tea Research Institute, Guizhou Academy of Agricultural Sciences, Guiyang 550006, Guizhou, China

## Abstract

Tea plant [*Camellia sinensis* (L.) O. Kuntze] is a globally important crop but is severely threatened by *Toxoptera aurantia* infestations, which impact yield and safety. However, the response of tea plants to aphid feeding remains largely unexplored. This study investigates the feeding behavior of *T. aurantia* on different cultivars and identifies ‘Huangjinya’ and ‘Qiancha 1’ as susceptible and resistant cultivars, respectively. Transcriptome analysis revealed that *CsUGT89A2* was significantly upregulated in response to *T. aurantia* infestation. *In vitro* biochemical assays demonstrated that *CsUGT89A2* encodes a flavonoid 7-glycosyltransferase that catalyzes the conversion of flavonoids and UDP-glucose into flavonoid 7-*O*-glucosides. *In vivo*, silencing *CsUGT89A2* significantly reduced flavonoid glycoside accumulation. To further clarify the role of *CsUGT89A2* in tea plant resistance to *T. aurantia*, we used tobacco and tea flowers to evaluate aphid feeding and reproduction under chemical treatment, gene silencing, and gene overexpression conditions. Statistical analysis showed that, compared with flavonoids, the application of flavonoid 7-*O*-glycosides significantly reduced *T. aurantia* reproductive capacity. Furthermore, compared with the control, overexpression of *CsUGT89A2* significantly reduced the reproductive ability of aphids, while its silencing increased reproductive rates. Overall, our findings demonstrate that *CsUGT89A2* mediates flavonoid glycosylation and enhances insect resistance in tea plants by increasing flavonoid glycoside levels, offering new insights into the role of flavonoid glycosides in the insect resistance of *C. sinensis*.

## Introduction

Plants and insects have co-evolved for billions of years, forming a complex network of offensive and defensive strategies. As immobile organisms, plants have developed a multilayered chemical defense system to resist herbivorous insect invasion, with the synthesis and modification of secondary metabolites serving as key pillars of this system [1]. Among these metabolites, flavonoids play a vital role in plant chemical defense owing to their structural diversity and multifunctionality. These polyphenolic compounds, derived from the phenylpropanoid pathway, are involved in physiological processes, such as flower coloration and UV protection, and also play dual roles in plant–insect interactions [2]. they can directly disrupt insect physiological metabolism and act as signaling molecules to activate systemic defense responses [3, 4]. However, free flavonoids in plants are easily degraded by environmental factors, and their lipophilicity limits their long-distance transport efficiency, prompting the evolution of glycosylation mechanisms to enhance their stability and bioavailability [5, 6]. The UDP-glucosyltransferase (UGT) family, key players in this process, catalyzes the transfer of glycosyl groups from activated donors to specific flavonoid sites, producing structurally stable and functionally active flavonoid glycosides. With advances in molecular biology, researchers have increasingly shown that UGT-mediated glycosylation not only serves as a regulatory hub for plant metabolic engineering [7, 8] but also represents a promising breakthrough for mechanistic insights.

The tea aphid (*Toxoptera aurantia* Boyer de Fonscolombe) is a globally important agricultural pest, known for rapid reproduction and large population sizes. As one of the most destructive agriculture pests, it affects not only tea plants but also citrus fruit, lychee, banana, and pineapple crops, among others [9]. It causes billions of dollars in agricultural losses worldwide annually [10, 11]. Tea aphids feed by sucking phloem sap from tender leaves and buds. During infestation, they secrete honeydew, which promotes fungal growth and spreads plant viruses [12, 13]. Affected leaves may curl and dry out, and in severe cases, plants may die. Although traditional chemical pesticides can control *T. aurantia* populations temporarily, issues of resistance evolution [14] and ecological risks [15] have driven research into intrinsic plant resistance mechanisms. In this context, the aphid-repellent function of flavonoid glycosides has garnered increasing attention. For example, exogenous gallic acid application has been shown to enhance direct defense responses against *Ectropis obliqua* in tea plants, significantly upregulating apigenin, naringin, and epigallocatechin 3-gallate expression levels [16]. Similarly, when tea plants are infected with gray blight, genes involved in flavonoid biosynthesis, such as chalcone synthase and phenylalanine lyase, are upregulated, along with elevated catechin concentrations in plant metabolites [17]. These findings suggest that plants form a dynamic defense network via UGT-regulated flavonoid glycosylation.

The UGT superfamily, as one of the largest enzyme systems in the plant kingdom, provides a molecular basis for the structural variation of flavonoid glycosides owing to its functional diversity [18]. It binds activated sugar molecules to acceptor substrates [19], and this specialized division of labor enables plants to produce flavonoid glycosides with various spatial conformations, targeting different insect physiological systems. In tobacco, UGT gene silencing markedly diminished resistance to *Myzus persicae*, highlighting the importance of these genes in plant defense against aphids [20]. Likewise, distinct gene expression in *Aphis nerii* on various toxic host plants indicates a detoxification role for UGT genes [21]. These findings suggest that plants form a dynamic defense network through UGT-regulated flavonoid glycosylation, although the underlying molecular mechanisms remain unclear.

The central challenge in the current research lies in elucidating the chemical structure–biological activity–ecological impact relationship. Although advances in biotechnology have allowed structural analysis of flavonoid glycosides, their dynamic distribution in living tissues and interactions with aphids remain difficult to observe in real time. This study is positioned at the intersection of plant chemical ecology and synthetic biology. Using molecular biology techniques, including multi-omics integration, gene silencing, overexpression, and exogenous application of *CsUGT89A2* enzyme products, this study systematically explores the UGT-mediated, flavonoid glycosylation–dependent aphid resistance mechanism. A model of flavonoid glycoside structural modification and its role in reducing aphid feeding was established for the first time, revealing how specific glycosylation sites influence variations in aphid feeding behavior.

## Results

### Transcriptome and differentially expressed gene analysis of tea plants after *T. aurantia* infestation

**Figure 1:**
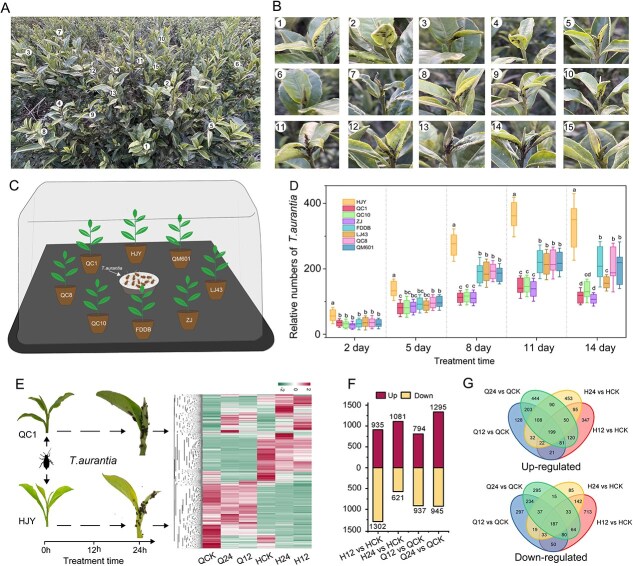
Evaluation and transcriptomic analysis of *T. aurantia* feeding preferences. (A, B) Field observations of *T. aurantia* damage to tea leaves in tea gardens. (C) Feeding preference experiment of *T. aurantia* among eight tea cultivars. (D) Population dynamics of *T. aurantia* across eight tea cultivars. Values represent the mean ± standard deviation, and different letters above the bars indicate significant differences (*P* < 0.05), determined by one-way ANOVA. (E) Heatmap showing the expression profiles of differentially expressed genes across six groups. (F) Number of upregulated and downregulated genes across four pairwise comparisons. (G) Venn diagrams showing the numbers of DEGs under *T. aruantii* infestation at different time points.

As piercing–sucking insects that infest the tender shoots of tea plants, *T. aurantia* pose a major threat to tea plantation productivity (Fig. 1A, B). To examine differences in *T. aurantia* feeding behavior among various tea varieties, eight cultivars with distinct leaf color traits were selected for infestation experiments, and *T. aurantia* population dynamics were monitored for each cultivar (Fig. 1C). Results showed that *T. aurantia* populations increased over time across cultivars, peaking on day 11. Notably, the chlorotic cultivar ‘Huangjinya’ (HJY) supported the highest population growth rate, while the green-leaf cultivar ‘Qiancha 1’ (QC1) and the purple-leaf cultivar ‘Zijuan’ exhibited the lowest rates (Fig. 1D). Based on these observations, HJY and QC1 were selected to explore the molecular mechanisms underlying differential aphid susceptibility. RNA sequencing (RNA-Seq) was performed on samples collected at 0, 12, and 24 h postinfestation (hpi; Fig. 1E). Detailed RNA-Seq results are presented in Table S1. Transcriptional profiling revealed significant changes in gene expression patterns in both cultivars following aphid infestation, as illustrated in hierarchical clustering heatmaps.

Differential expression analysis identified 2237 and 1702 differentially expressed genes (DEGs) at 12 hpi (H12 vs. HCK) and 24 hpi (H24 vs. HCK), respectively, whereas QC1 showed 1731 and 2240 DEGs at 12 hpi (Q12 vs. QCK) and 24 hpi (Q24 vs. QCK), respectively (Fig. 1F). Venn diagram analysis revealed 199 and 187 co-upregulated and co-downregulated genes, respectively, common to all four comparisons (Fig. 1G), which were prioritized for further functional analysis.

### *LOC114303213* response to *T. aurantia* based on Gene ontology and Kyoto encyclopedia of genes and genomes analyses

**Figure 2:**
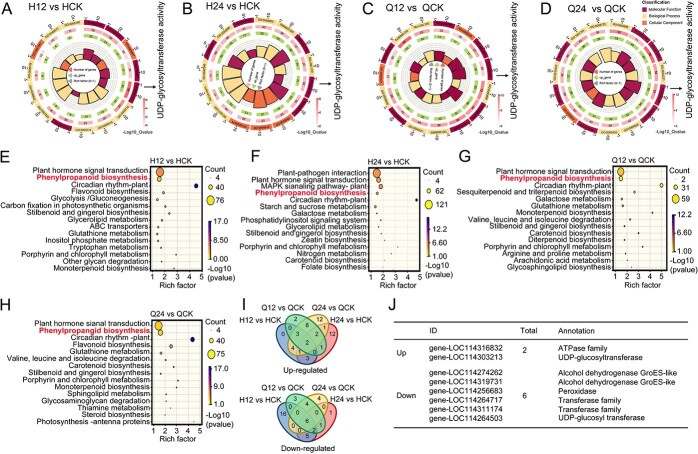
Transcriptional response of tea plants to *T. aurantia* feeding. (A–D) Top 15 significantly enriched GO terms among DEGs in each pairwise comparison. Complete GO term names are provided in Table S2. (E–H) Top 15 enriched KEGG pathways among DEGs. (I) Venn diagram showing overlap of DEGs involved in the phenylpropanoid biosynthetic pathway. (J) Functional annotation of upregulated and downregulated genes within the phenylpropanoid pathway.

To characterize the functional roles and metabolic pathway changes of DEGs in tea plants under *T. aurantia* infestation, Gene Ontology (GO) and Kyoto Encyclopedia of Genes and Genomes (KEGG) enrichment analyses were performed. GO analysis identified three biological processes consistently enriched across all treatment groups (H12 vs. HCK, H24 vs. HCK, Q12 vs. QCK, and Q24 vs. QCK): transcription factor activity, sequence-specific DNA binding, and oxidoreductase activity (Fig. 2A–D; Table S2). KEGG analysis revealed significant enrichment of pathways associated with phenylpropanoid biosynthesis, plant hormone signal transduction, and carotenoid biosynthesis in the same comparisons (Fig. 2E–H). Phenylpropanoids, key secondary metabolites involved in plant stress responses, are widely recognized for their roles in mediating resistance to biotic and abiotic stressors [22, 23]. Prior studies on *T. aurantia* interactions have also implicated the phenylpropanoid pathway in tea plant defense against aphid-induced stress [24]. To further examine this relationship, co-expression clustering analysis of phenylpropanoid-related DEGs was conducted across the four treatment groups (Fig. 2I). Results revealed consistent upregulation of two genes and downregulation of six genes under all infestation conditions. Notably, the co-upregulated genes *LOC114316832* (encoding an ATPase family protein) and *LOC114303213* (encoding a UDP-glucosyltransferase) exhibited significant induction levels (6.4-fold and 14.8-fold, respectively; Fig. 2J). *LOC114303213*, annotated as a glycosyltransferase, was associated with both GO terms and KEGG pathways, highlighting its potential regulatory role in tea plant defense responses to *T. aurantia.*

### *CsUGT89A2* responds to biological stress induction

**Figure 3:**
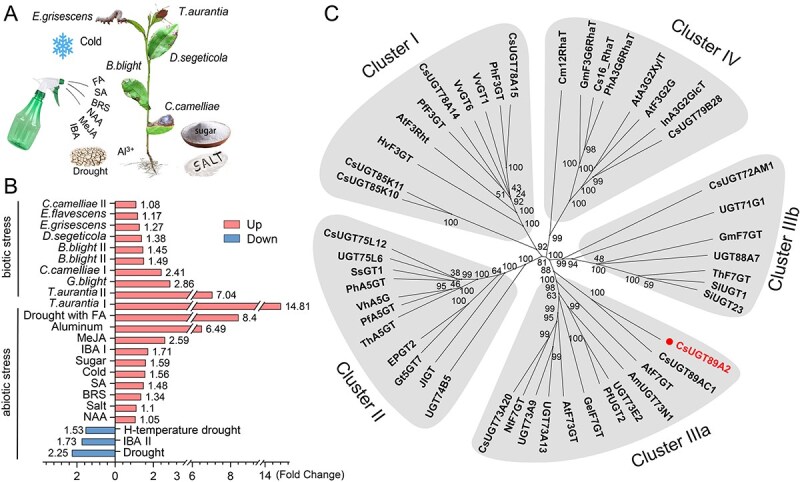
Stress-responsive expression and phylogenetic analysis of *CsUGT89A2.* (A) Schematic illustrating 23 distinct stress treatments applied to tea plants, including biotic and abiotic stressors. (B) Heatmap showing transcript levels of *CsUGT89A2* under the 23 stress conditions. (C) Phylogenetic tree of *CsUGT89A2* and functionally characterized UGTs from *Camellia* and related species. GenBank accession numbers used in the phylogenetic analysis are listed in Table S3.

To determine whether *LOC114303213* is involved in other stress responses in tea plants, its expression profile was analyzed using transcriptome data from 23 distinct stress conditions (Fig. 3A). Expression analysis showed that *LOC114303213* was upregulated and downregulated under 20 and 3 stress conditions, respectively (Fig. 3B). Among the upregulated responses, 10 corresponded to biotic stressors (e.g., *T. aurantia* infestation) and 10 to abiotic stressors (e.g., drought). The most pronounced upregulation occurred under *T. aurantia* I (‘Qiancha 1’ group: 14.81-fold), drought combined with fulvic acid (8.41-fold), and *T. aurantia* II (‘Huangjinya’ group: 7.04-fold). All downregulated responses were linked to abiotic stressors.

To infer the biological role of *LOC114303213* (designated *CsUGT89A2*), a phylogenetic tree was constructed using functionally characterized UGT genes from tea plants (Fig. 3C). The tree clustered genes into five clades (I, II, IIIa, IIIb, and IV), corresponding to glycosyltransferases with distinct catalytic specificities: 3-*O*, 5-*O*, 7-*O*, and branched glycosylation [25]. *CsUGT89A2* and *UGT89AC1* clustered within the 7-*O*-glycosyltransferase group. *UGT89AC1* is known to mediate quercetin glycoside biosynthesis and contributes to resistance against *Ectropis grisescens* infestation [26]. Although the function of *CsUGT89A2* remains uncharacterized, integrated analysis of gene expression and phylogeny suggests that it plays a similar role to *UGT89AC1*, potentially enhancing flavonoid glycosylation to mitigate *T. aurantia*-induced stress.

### *In vitro*, rCsUGT89A2 catalyzes the biosynthesis of flavonoid 7-*O*-glucosides

**Figure 4:**
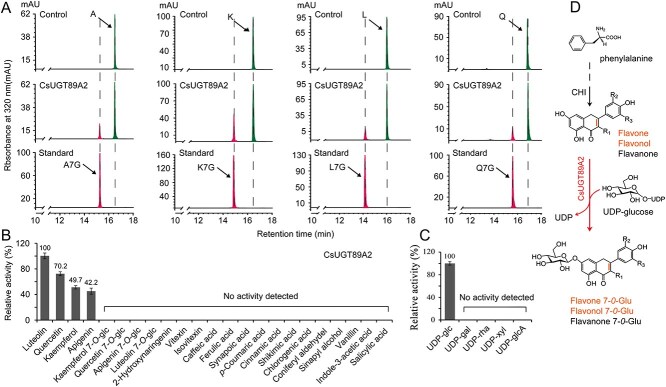
HPLC analysis of recombinant CsUGT89A2 enzymatic activity. (A) HPLC chromatogram showing products derived from UDP-glucose (UDP-Glu) conversion by recombinant CsUGT89A2. (B , C) Substrate specificity analysis of recombinant CsUGT89A2. Panels B and C show results for flavonoid aglycones and sugar donors, respectively. (D) Proposed biosynthetic role of CsUGT89A2 in catalyzing flavonoid 7-*O*-glycoside formation.

To validate the catalytic role of CsUGT89A2 in flavonoid glycosylation, recombinant CsUGT89A2 protein (rCsUGT89A2) was purified using Ni-TED affinity chromatography, yielding a single 53.6-kDa band on SDS-PAGE (Fig. S1), confirming high purity for enzymatic assays. Based on functional predictions, a diverse panel of natural secondary metabolites and sugar donors were selected to evaluate the recombinant protein’s enzymatic activity *in vitro*. Specifically, rCsUGT89A2 catalyzed A-7-G, Q-7-G, K-7-G, and L-7-G formation from apigenin, quercetin, kaempferol, and luteolin, respectively, using UDP-glucose as the sugar donor (Figs 4A and S2). No activity was detected when using alternative donors (UDP-rhamnose, UDP-galactose, UDP-xylose, or UDP-glucuronic acid) or nonflavonoid acceptors (phenolic acids or lignin precursors) (Fig. 4B). Kinetic assays confirmed that rCsUGT89A2 exclusively utilized UDP-glucose to catalyze 7-*O*-glucosylation of flavonoid substrates (Fig. 4C). These findings demonstrate that rCsUGT89A2 is an UDP-glucose-dependent glucosyltransferase with strict regioselectivity for the 7-hydroxyl position of flavonoids and broad acceptor specificity (Fig. 4D).

### Biochemical characterization and kinetic parameters of rCsUGT89A2

**Figure 5:**
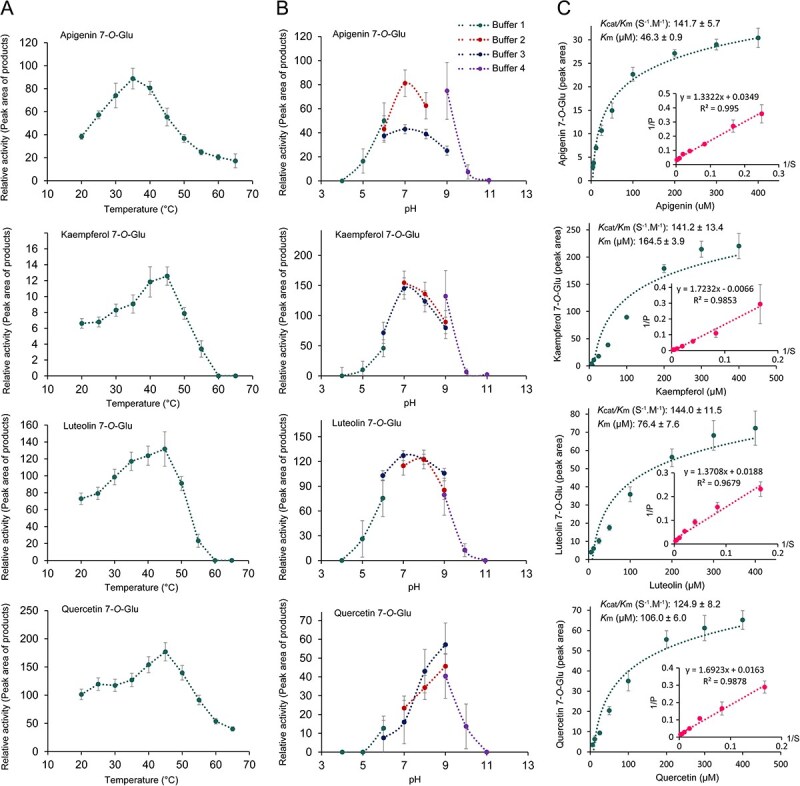
Optimization of catalytic conditions and kinetic analysis of recombinant CsUGT89A2. (A) Optimal reaction temperature (20–65°C) for the glycosylation activity of CsUGT89A2 on four different substrates, measured at pH 8.0. (B) Optimal pH range (4.0–11.0) for the glycosylation activity of CsUGT89A2 on four different substrates, measured at 40°C. (C) Kinetic parameters of recombinant CsUGT89A2, measured at pH 8.0 and 40°C. Data are presented as means ± standard errors from three replicates.

To characterize the enzymatic properties of rCsUGT89A2, its catalytic efficiency was evaluated under varying reaction temperatures and pH conditions. Optimal activity was observed at 35–45°C for all four flavonoid substrates (Fig. 5A). Additionally, pH profiling (4.0–11.0) revealed substrate-dependent optima (Fig. 5B): kaempferol and luteolin exhibited peak activity at pH 7.0–8.0, quercetin at pH 8.0–9.0, and apigenin demonstrated broader stability at pH 7.0–9.0. Enzymatic activity remained robust across most tested conditions. Kinetic analysis revealed distinct substrate affinities (Fig. 5C), with *K*m values of 46.3 μM (apigenin), 76.4 μM (luteolin), 164.5 μM (kaempferol), and 106.0 μM (quercetin), respectively. Higher catalytic efficiency (*Kcat*/*K*m) for apigenin and luteolin compared with kaempferol and quercetin indicated a preference for these substrates.

### *In vivo, CsUGT89A2* and its catalyzed flavonoid 7-*O*-glucoside enhance tobacco resistance to *M. persicae*

**Figure 6:**
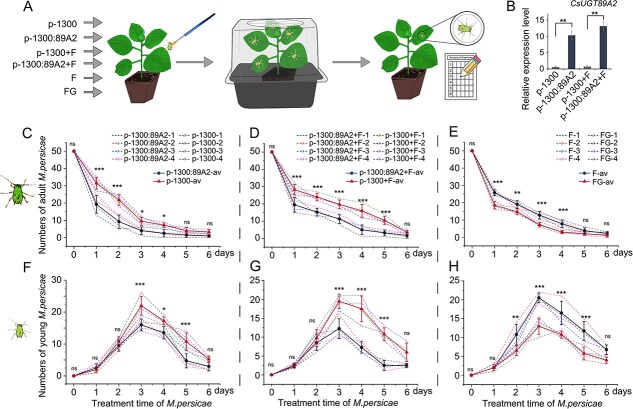
Impact of heterologous *CsUGT89A2* expression and flavonoid treatment on aphids in tobacco (*Nicotiana tabacum*). (A) Schematic diagram of tobacco infection with *M. persicae* following various treatments. (B) Relative expression levels of *CsUGT89A2* under different experimental conditions. (C, F) Effects of CsUGT89A2 overexpression versus the empty vector on adult and nymph *M. persicae* populations. (D, G) Effects of CsUGT89A2 overexpression versus the empty vector with exogenous flavonoid aglycone treatment on adult and nymph *M. persicae* populations. (E, H) Effects of flavonoid aglycones versus the corresponding flavonoid glycosides on adult and nymph *M. persicae* populations. Dashed lines represent different individual replicates; solid lines show means ± standard errors of four replicates. Statistical differences were determined using two-way ANOVA in SPSS 27.0 (*P < 0.05, **P < 0.01, and ***P <0.001). “av” denotes the average of replicates.

To investigate the physiological roles of *CsUGT89A2* and its product flavonoid 7-*O*-glucoside (FG) in plant defense, transgenic tobacco lines overexpressing *CsUGT89A2* and FG-treated plants were subjected to aphid bioassays (Fig. 6A). Importantly, qRT-PCR confirmed significant upregulation of *CsUGT89A2* in ‘p-1300: *CsUGT89A2*’ and ‘p-1300: *CsUGT89A2* + F’ compared with controls (‘p-1300’ and ‘p-1300 + F’; Fig. 6B). Adult aphid densities declined in all groups postinoculation, with markedly lower densities observed in *CsUGT89A2*-overexpressing (p-1300: *CsUGT89A2*; p-1300: *CsUGT89A2* + F) and FG-treated plants relative to controls (p-1300, p-1300 + F, and F) (Fig. 6C–E). Similarly, nymphal populations initially increased before declining, with consistently reduced densities observed in treatment groups compared to controls (Fig. 6F–H). These results, together with biochemical data, demonstrate that *CsUGT89A2* enhances aphid resistance by catalyzing flavonoid glycosylation, with FG accumulation acting as the primary biochemical defense mechanism in transgenic hosts.

### Functional validation of *CsUGT89A2* through gene silencing in tea plants

**Figure 7:**
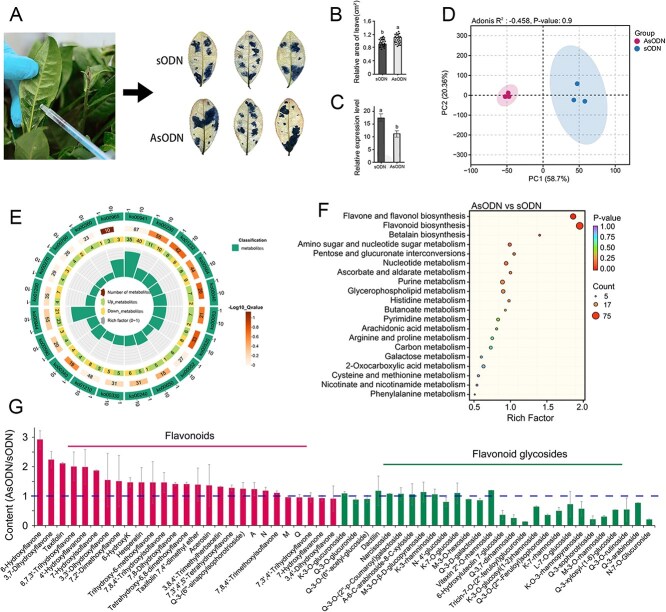
Metabolic changes in tea leaves following *CsUGT89A2* gene silencing. (A) Changes in NBT staining intensity in tea leaves after gene silencing. (B) Quantification of NBT-stained areas. (C) Relative expression levels of *CsUGT89A2* post silencing. (D) PCA comparing AsODN-*CsUGT89A2* and sODN treatments. (E, F) KEGG pathway enrichment analysis of DEGs from AsODN-CsUGT89A2 versus sODN. (G) Differential flavonoid metabolism profiles between AsODN-*CsUGT89A2* and sODN. Data are presented as means ± standard errors from three biological replicates.

Owing to the lack of a stable and efficient genetic transformation in tea plants, antisense oligodeoxynucleotide (AsODN)-mediated transient gene silencing, a rapid and effective method validated in tea plants [27], was employed to investigate *CsUGT89A2* function (Fig. 7A). Comparative analyses of AsODN-treated and sense ODN (sODN) control plants included nitroblue tetrazolium (NBT) staining, qRT-PCR analysis, and metabolomic profiling. NBT staining revealed a 1.4-fold increase in reactive oxygen species accumulation in *CsUGT89A2*-silenced leaves relative to controls (Fig. 7B), suggesting impaired antioxidant capacity is linked to altered flavonoid metabolism [28]. qRT-PCR confirmed successful silencing, with significantly reduced *CsUGT89A2* transcript levels observed in AsODN-treated samples (Fig. 7C).

Principal component analysis (PCA) of secondary metabolites revealed clear metabolic divergence between groups, with principal component (PC)1 and PC2 explaining 58.70% and 20.36% of the total variance, respectively (Fig. 7D). KEGG pathway enrichment of differentially accumulated metabolites identified enrichment in the anthocyanin biosynthesis (ko00942), flavone and flavonol biosynthesis (ko00944), isoquinoline alkaloid biosynthesis (ko00950), and glucosinolate biosynthesis (ko00966) pathways (Fig. 7E, F; Table S3). Metabolite quantification showed elevated free flavonoid levels and reduced flavonoid glycoside content in AsODN-treated plants relative to controls (Fig. 7G).

### Exogenous application of flavonoid glycosides inhibits *T. aurantia* activity in tea plants

**Figure 8:**
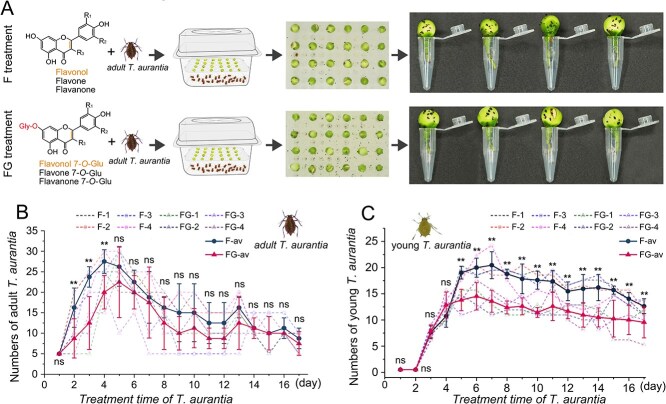
Effects of exogenous flavonoid application on *T. aurantia*. (A) Schematic representation of *T. aurantia* treatments using flavonoid aglycones and glycosides. (B) Population dynamics of adult *T. aurantia* over 17 days following treatment. (C) Population dynamics of nymphs over the same period. Colored dashed lines indicate biological replicates; solid lines show the means ± standard errors of four replicates. Two-way ANOVA was performed in SPSS 27.0 to determine significant differences between the treatment and control groups (*P < 0.05, **P < 0.01, and ***P < 0.001).“F” refers to the flavonoid aglycone mixture, whereas “FG” denotes the flavonoid glycoside mixture. “av” denotes the average of replicates.

Although flavonoid glycosides have shown inhibitory effects on *M. persicae*, their role in tea plant–*T. aurantia* interactions remains unclear. To evaluate their potential in modulating *T. aurantia* populations, exogenous flavonoid glycosides were applied to tea plants, and aphid population dynamics were monitored (Fig. 8A). During the first 4 days post–treatment, adult and nymph population growth trends were similar between the flavonoid glycoside–treated and aglycone flavonoid control groups (Fig. 8B, C). However, by day 5, both groups exhibited declining aphid numbers, with the flavonoid glycoside-treated group showing a markedly slower reduction rate. Throughout the experimental period, *T. aurantia* population densities remained consistently lower in glycoside-treated plants compared with controls. These findings suggest that flavonoid glycosylation enhances chemical defense against *T. aurantia* in tea plants. Glycosylated derivatives likely confer improved phytochemical stability and bioavailability over aglycones, enabling a biphasic defense response: an initial activation (days 1–4) followed by sustained feeding deterrence and population suppression (days 5–7). This metabolic transformation may extend the bioactive lifespan of flavonoids, contributing to reduced aphid viability and population persistence.

### ***CsUGT89A2*** s**ilencing increases the reproductive capacity of *T. aurantia***

**Figure 9:**
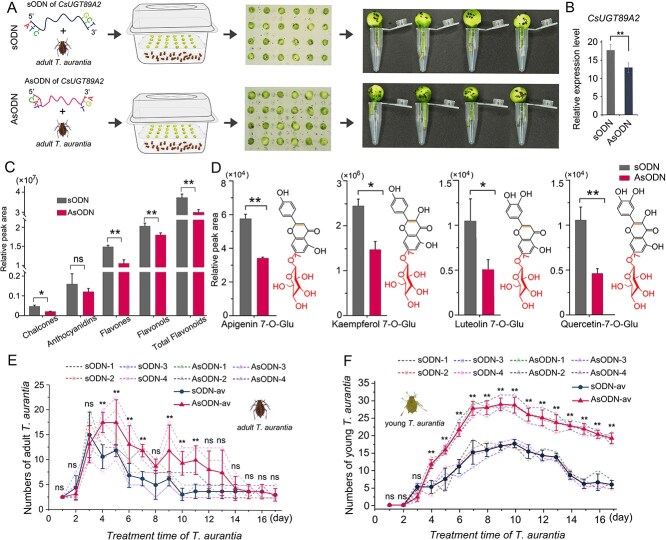
Effects of *CsUGT89A2* gene silencing on *T. aurantia* behavior and tea flower metabolites. (A) Schematic of aphid inoculation following AsODN or sODN treatment. (B) Relative expression levels of *CsUGT89A2* in silenced versus control tea flowers. (C) Relative peak areas of key flavonoids following gene silencing. (D) Relative peak areas of apigenin 7-*O*-glucoside, kaempferol 7-*O*-glucoside, luteolin 7-*O*-glucoside, and quercetin 7-*O*-glucoside after gene silencing. (E) Population trend of adult *T. aurantia* over 17 days following AsODN-CsUGT89A2 and sODN treatment. (F) Population trend of *T. aurantia* nymphs over the same period. (B–D) Data represent means ± standard errors of three biological replicates, with one-way ANOVA used to determine significant differences between groups. (E, F) Dashed lines indicate biological replicates (*n* = 4); solid lines denote means ± standard errors. Two-way ANOVA was employed to determine significant differences between the treatment and control groups (*P < 0.05, **P < 0.01, and ***P < 0.001).“av” denotes the average of replicates.

Although transient overexpression of *CsUGT89A2* in tobacco-enhanced resistance to *M. persicae*, its functional role in tea plant defense against *T. aurantia* remained unverified. To address this, AsODN-mediated *CsUGT89A2* silencing was employed in tea plants, and aphid population dynamics were analyzed (Fig. 9A). qPCR confirmed effective suppression of *CsUGT89A2* expression in silenced plants compared with sODN controls (Fig. 9B). Metabolic analysis showed significantly decreased levels of multiple flavonoid glycosides in *CsUGT89A2*-silenced tea flowers (Fig. 9C; Table S6), including the four 7-*O*-glucosides previously identified as enzymatic products of rCsUGT89A2, significantly downregulated (Fig. 9D; Table S6). Following aphid inoculation, adult *T. aurantia* populations in both groups increased until day 3, followed by declines from day 4 onward; however, population reduction was slower in the silencing group (Fig. 9E). Nymph counts in the *CsUGT89A2*-silenced group surpassed those in controls by day 4 and remained elevated until day 9 before declining (Fig. 9F). These results indicate that *CsUGT89A2* silencing weakens tea plant resistance by diminishing feeding deterrence and reproductive suppression of *T. aurantia*. Taken together with *in vitro* enzymatic data, these findings confirm that *CsUGT89A2*-mediated biosynthesis of flavonoid 7-*O*-glucosides contributes markedly to the phytochemical defense of tea plants, underscoring its central role in *T. aurantia* resistance.

## Discussion

### Effects of *T. aurantia* infestation on phenylpropanoid and flavonoid biosynthesis pathways

The development, survival, and reproduction of herbivorous insects are strongly influenced by the type and concentration of host plant secondary metabolites [29, 30]. In response to insect herbivory, plants activate defense-signaling cascades that induce downstream stress-responsive genes and initiate defense mechanisms [31]. Previous studies on insect-induced plant stress responses have primarily focused on physiological and biochemical parameters. Among plant secondary metabolic pathways, phenylpropanoid and flavonoid biosynthesis are known to play critical roles in biotic stress resistance. Transcriptomic and metabolomic analyses have shown that flavonoid compounds contribute to pathogen resistance against powdery mildew in melon plants [32], and sugarcane upregulates phenylpropanoid and flavonoid-related genes in response to armyworm (*Mythimna separata*) herbivory [33].

In preliminary investigations into tea plant responses to *T. aurantia* infestation, resistant (W016) and susceptible (HJY) cultivars were compared, revealing that this pest challenge activates phenylpropanoid metabolism [24]. To further evaluate the molecular mechanisms underlying phenylpropanoid-mediated defense against *T. aurantia*, transcriptomic profiling of the susceptible HJY and resistant QC1 cultivars was conducted. DEG analysis across four comparison groups revealed pronounced enrichment of phenylpropanoid and flavonoid biosynthesis pathways in infested plants [34]. Notably, these metabolites also contribute to tea flavor, imparting its characteristic sensory qualities [35]. Given the pleiotropic roles of phenylpropanoid pathways in plant stress adaptation and product quality, targeted modulation of gene expression and metabolite flux within this pathway may represent a viable strategy for mitigating *T. aurantia-*induced damage in tea crops.

### Flavonoid glycosides suppress *T. aurantia* reproduction

Flavonoids are a major class of secondary metabolites typically found in glycosylated forms, which confer enhanced chemical stability, water solubility, bioactivity, and pharmacokinetic properties relative to their aglycone counterparts, facilitating their participation in plant defense against biotic and abiotic stressors [36]. The critical role of flavonoids in mediating plant resistance to insect herbivory is well established [37, 38]. For instance, they function as signaling molecules to activate defense cascades as well as antifeedants or toxins against pests [39]. Glycosylated derivatives of quercetin and naringin have been linked to stress resilience in *Bombyx mori*, and infestation by *Tetranychus urticae* upregulates genes associated with hydroxyflavone, naringenin, and hesperetin biosynthesis [40, 41].

In this study, a *T. aurantia* reproductive capacity assay revealed that exogenous flavonoid glycoside application significantly reduced *T. aurantia* fecundity relative to flavonoid aglycone application alone. This is consistent with prior studies reporting that glycosylated flavonoids interfere with insect larval metamorphosis, thereby suppressing reproductive success [42]. Collectively, these findings underscore the pivotal role of flavonoid glycosylation in plant defense. Based on this evidence, we hypothesize that *T. aurantia* infestation induces flavonoid glycoside biosynthesis in tea plants, enhancing resistance to phytophagous *T. aurantia* populations.

### *CsUGT89A2* responds to *T. aurantia* by regulating the balance between flavonoids and flavonoid glycosides

UGTs are key enzymes involved in plant development and stress adaptation through the glycosylation of secondary metabolites [43–46]. For example, UGTs in wheat (*Triticum aestivum*) enhance resistance to *Fusarium* head blight and *Magnaporthe oryzae* [47, 48]. In *C. sinensis*, UGTs catalyze flavonoid and phytohormone glycosylation, contributing to defense mechanisms targeting herbivores such as *E. obliqua* and *Spodoptera litura* [26, 49, 50].

The present study examined the stress-induced expression of *CsUGT89A2* in tea plants. Transcriptomic profiling showed significant upregulation of *CsUGT89A2* under biotic stress, including infection by fungal pathogens (e.g. *Didymella segeticola*, blister blight, and *Colletotrichum camelliae*). Notably, *T. aurantii* infestation elicited the most pronounced response, with *CsUGT89A2* transcript levels increasing 7.04-fold in the susceptible HJY and 14.81-fold in the moderately resistant QC1 compared with controls. *In vitro* enzymatic assays confirmed *CsUGT89A2*’s capacity to catalyze flavonoid glycosylation. To assess its functional role in resistance, RNA interference–mediated silencing was performed, followed by *T. aurantia* reproductive capacity assays. Silencing *CsUGT89A2* markedly reduced flavonoid glycoside levels and led to a corresponding increase in *T. aurantia* fecundity.

**Figure 10:**
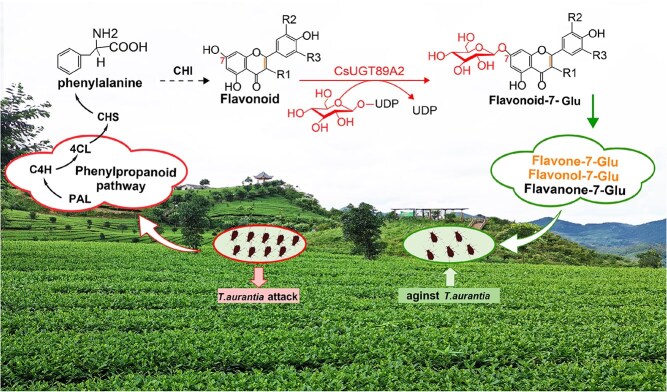
Proposed model of *T. aurantia–induced CsUGT89A2* activation and its role in antiherbivore defense. Feeding by *T. aurantia* induces sustained expression of *CsUGT89A2*, which facilitates position-specific 7-*O*-glycosylation of flavonoid scaffolds, enhancing the tea plant’s defense against subsequent *T. aurantia* attacks.

Overall, this study identified *CsUGT89A2* as a glycosyltransferase gene showing strong upregulation in response to aphid infestation. Functional characterization demonstrated that the enzyme catalyzes 7-*O*-glycosylation of various flavonoid aglycones, generating corresponding flavonoid 7-*O*-glycosides. Moreover, heterologous overexpression in both tea and tobacco model systems confirmed its role in augmenting plant resistance to aphid infestation. Based on these findings, we propose a mechanistic model in which *CsUGT89A2*-mediated glycosylation regulates flavonoid glycoside biosynthesis as a coordinated defense strategy against phytophagous insect stress (Fig. 10).

## Materials and methods

### Plant materials

Nine cultivars of three-year-old tea plants (*C. sinensis*) at the vegetative growth stage were used as experimental materials, including ‘Huangjinya’ (HJY), ‘Qiancha 1’ (QC1), ‘Fuding Dabaicha’ (FDDB), ‘Zijuan’ (ZJ), ‘Qiancha 8’ (QC8), ‘Qiancha 10’ (QC10), ‘Qianmei 601’ (QM601), ‘Shuchazao’ (SCZ), and ‘Longjing 43’ (LJ43). Additionally, unopened floral buds were collected from ‘Shuchazao’ at the reproductive stage. All tea plant materials were obtained from the Tea Research Institute of the Guizhou Academy of Agricultural Sciences. To ensure consistency, tea plants were grown under controlled conditions: 25 ± 2°C, 70 ± 5% relative humidity, and a 16/8-h light/dark photoperiod. For comparative analysis, tobacco (*Nicotiana tabacum*) plants at the vegetative stage, provided by College of Tea Science, Guizhou University, were cultivated under identical environmental conditions.

### Insect material and host selection assay

Both *T. aurantia* and *M. persicae* were obtained from College of Tea Science, Guizhou University and maintained for over three generations under the conditions described in Section 4.1. To evaluate insect resistance among tea cultivars, six green (QC1, FDDB, QC8, QC10, QM601, LJ43), one chlorotic (HJY), and one purple cultivar (ZJ) were selected for aphid feeding preference assays. Potted seedlings were arranged in a circle, and wingless adult aphids were starved for 1 h before being placed in a Petri dish at the center of the arena for host selection. Four independent experimental replicates were conducted within insect-proof nets (1 × 1 × 0.8 m) to minimize external interference. Aphid populations on each cultivar were recorded on days 2, 5, 8, 11, and 14 postinfestation.

### Tea plant cultivar selection and *T. aurantia* treatment

Based on the aphid host selection assay, HJY and QC1, cultivars exhibiting significant differences in *T. aurantia* infestation, were selected. Thirty wingless adult aphids were starved for 1 h before the experiment, before being placed on the tea shoots of HJY and QC1. Feeding durations were 0 h (HCK and QCK), 12 h (H12 and Q12), and 24 h (H24 and Q24), creating six treatment groups. Each treatment group was replicated five times. After treatment, three seedlings per group were randomly selected. The apical parts (one bud and two leaves) were carefully wrapped in aluminum foil, frozen in liquid nitrogen, and stored at −80°C for further analysis.

### RNA-Seq

Total RNA was extracted from HCK, QCK, H12, Q12, H24, and Q24 samples using FastPure Universal Plant Total RNA Isolation Kit (Vazyme, Ltd., Nanjing, China) following the manufacturer’s instructions. RNA purity and concentration were measured using a NanoDrop 2000 spectrophotometer (Wilmington, DE, USA). A cDNA library was constructed using the TruSeq Strand mRNA LT Sample Prep Kit (San Diego, CA, USA) and sequenced on the Illumina NovaSeq 6000 platform by Biomarker Technologies Co., Ltd. (http://www.biomarker.com.cn/, Beijing, China). Transcriptome data were aligned to the *C. sinensis* genome (https://ngdc.cncb.ac.cn/gwh/Assembly/display/12830). Gene expression was quantified as fragments per kilobase of transcript per million fragments. DEGs were identified using DESeq2 with a fold change ≥1.5 and *P* < 0.05. GO (http://geneontology.org/) and KEGG (https://www.genome.jp/kegg/) enrichment analyses were performed using the clusterProfiler packages.

### Gene expression profiling and phylogenetic analysis of UGTs

RNA-Seq datasets from 21 distinct stress conditions were sourced from NCBI (https://www.ncbi.nlm.nih.gov) and China National Center for Bioinformation (CNCB) (https://bigd.big.ac.cn/gsa). These datasets are *T. aurantii* I, *T. aurantii* II, *Colletotrichum camelliae* (*C. camelliae*) I, *C. camelliae* II, Blister blight disease (B. blight) I, B. blight II, *Empoasca flavescens* (*E. flavescens*), *Ectropis grisescens* (*E. grisescens*), *Didymella segeticola* (*D. segeticola*), Gray blight and abiotic stresses Drought, IBA I, IBA II, High-temperature drought, NAA, Salt, BRS, SA, Cold, Sugar, MeJA, Aluminum, Drought with FA (The project numbers for these datasets can be found in the Data Availability Statement section.) At the same time, UGT genes identified in tea plants were screened, and their protein sequences were used for phylogenetic analysis with 1000 bootstrap replicates in MEGA 11.0, using the neighbor-joining method. GenBank accession numbers for all genes used in the study are listed in Table S4.

### Expression vector pRSFDuet-*CsUGT89A2*

The *CsUGT89A2* cDNA coding sequence was amplified using PrimeSTAR HS DNA Polymerase (Takara Bio Inc., Beijing, China). PCR products were separated via 1.2% (w/v) agarose gel electrophoresis and purified using a DNA gel extraction kit (Aidlab Co., Beijing, China). Purified DNA fragments were cloned into the pEASY-Blunt vector (TransGen Biotech, Beijing, China) and transformed into *E. coli* DH5α (TransGen Biotech, Beijing, China) for sequencing. The target plasmid was isolated using a high-purity plasmid microkit (Aidlab Co., Beijing, China). Both the target gene and pRSFDuet-1 vector were digested with BamHI, purified (Yeason Biotech Co., Shanghai, China), and ligated to produce pRSF-*CsUGT89A2* with a His tag. After sequencing confirmation, the plasmid was transformed into *E. coli* BL21(DE3) cells (TransGen Biotech, Beijing, China).

Precultured cells (OD_600_ ≈ 0.6–0.8) were induced with 0.5 mM IPTG at 28°C for 25 h to express rCsUGT89A2. Cells were then centrifuged at 12 000 × *g* for 15 min, and recombinant protein was purified using amylose resin via affinity chromatography. Protein purity was confirmed via SDS-PAGE. rCsUGT89A2 was further purified using linear starch resin and subjected to enzymatic assays.

### Chemicals and reagents

Kaempferol (K), quercetin (Q), luteolin (L), apigenin (A), kaempferol 7-*O*-glucoside (K-7-G), kaempferol 3-*O*-glucoside (K-3-G), quercetin 3-*O*-glucoside (Q-3-G), quercetin 7-*O*-glucoside (Q-7-G), apigenin 5-*O*-glucoside (A-5-G), apigenin 7-*O*-glucoside (A-7-G), luteolin 7-*O*-glucoside (L-7-G), UDP-glucose, UDP-rhamnose, UDP-galactose, and UDP-glucuronic acid were procured from Yuanye BioTechnology Co., Ltd. (Shanghai, China). Methanol, acetonitrile, and acetic acid were acquired from Shanghai Aladdin Biochemical Technology Co., Ltd. and utilized for examination via a reverse-phase high-performance liquid chromatography (HPLC) system.

### Enzymatic assays and kinetic parameter analyses

*In vitro* enzyme activity of rCsUGT89A2 was assessed using a 50-μL reaction system containing Tris–HCl buffer (100 mM, pH 8.0), containing 50 μL mixtures of 2.5 mM sugar donor, 0.3 mM sugar acceptor, and 100 μg of protein. Optimal pH and temperature parameters were determined using four buffers. Buffer 1: 100 mM sodium citrate (pH 4.0–6.0), Buffer 2: 100 mM Tris–HCl (pH 7.0–9.0), Buffer 3: 100 mM phosphate (pH 6.0–9.0), Buffer 4: 100 mM Na_2_CO_3_/NaHCO_3_ (pH 9.0–11.0). The temperature range was 20–65°C, and pH was tested at each unit. For kinetic assays, 50-μL reactions included 10 μg of protein, 2.5 mM UDP-glucose, and 0–400 μM sugar acceptors. Reactions were incubated at 40°C for 40 min in a water bath, terminated with methanol, and centrifuged for supernatant collection.

### HPLC and UHPLC-QQQ-MS/MS analysis

The activity of rCsUGT89A2 was assessed utilizing reverse-phase (HPLC) (Agilent Technologies, Santa Clara, CA). HPLC column: Venusil XBP C18 column (particle size: 5 μm; dimensions: 4.6 × 100 mm; utilized for detecting the enzymatic activity of rCsUGT89A2). The mobile phase consists of a 1% acetic acid aqueous solution (A) and 100% acetonitrile (B), with a flow rate of 0.6 mL min^−1^. The elution gradient is delineated as follows: 1% B for 0–10 min, 1–30% B for 10–20 min, 30–50% B for 20–24 min, 50–80% B for 24–26 min, 80–20% B for 26–30 min, and 20–1% B for 30–35 min. The detecting wavelength range is 270–350 nm.

The products of the enzymatic process were identified through the UHPLC-QQQ-MS/MS system. Chromatographic conditions: an ultrahigh-performance liquid chromatograph was implemented on a 1290 Infinity II series. The solvent system consisted of 0.4% (v/v) acetic acid (solvent A) and 100% acetonitrile (solvent B). Equilibration was conducted using the following gradient solvent system: 1–25% B for 0–5 min, 25–45% B for 5–14 min, 45–60% B for 14–22 min, 60–1% B for 22–24 min, and 1–1% B for 24–25 min. The solvent flow rate was 0.2 mL min^−1^. Mass spectrometer conditions: an Agilent 6460c triple quadrupole mass spectrometer (Agilent Technologies). Equipped with an electrospray ionization interface was applied to perform mass spectrometry in multiple reaction monitoring modes.

### Chemical application in tobacco leaves and *M. persicae* treatment

Flavonoid and glycoside mixtures at 0.03 mM were prepared using apigenin, kaempferol, luteolin, and quercetin, and their corresponding glycosides (A-7-G, K-7-G, L-7-G, and Q-7-G, respectively) to characterize *CsUGT89A2* function *in vivo*. Mixtures were injected into the abaxial side of mature tobacco leaves using a sterile syringe. Each treatment was replicated five times, and the injection volume ensured complete leaf infiltration. Plants were allowed to recover for 24 h under normal conditions (25°C with 16 h of light and 8 h of darkness). Subsequently, 10 wingless *M. persicae* adults were inoculated onto five middle leaves per plant. Aphid populations were monitored every 24 h.

### Transient overexpression of *CsUGT89A2* in tobacco facilitated by *Agrobacterium*

*CsUGT89A2* was cloned into *Agrobacterium GV3101* harboring the pCAMBIA1300 construct using gene-specific primers. Following PCR-based screening, positive transformants were inoculated into LB broth at 28°C until OD_600_ ≈ 0.6 was reached. Subsequently, bacterial cultures were harvested, centrifuged at 5000 × *g* and 4°C for 10 min, washed twice with sterile water, and resuspended in 10 mM MES buffer (pH 5.6) to OD_600_ ≈ 0.8. A flavonoid mixture (0.03 mM) was added to both pCAMBIA1300 and pCAMBIA1300-*CsUGT89A2* bacterial suspensions, creating two experimental groups. Four treatment groups were established: pCAMBIA1300 empty vector (p-1300), pCAMBIA1300-*CsUGT89A2* (p-1300: *CsUGT89A2*), the flavonoid mixture with p-1300 (p-1300 + F), and the flavonoid mixture with p-1300: *CsUGT89A2* (p-1300: *CsUGT89A2* + F). Solutions were infiltrated into mature tobacco leaves with four biological replicates per treatment. Plants were kept in darkness for 48 h, followed by recovery under standard conditions for 24 h. Post recovery, five plants were selected for aphid inoculation, and five middle leaves per plant were inoculated with 10 wingless *M. persicae* adults using a fine brush. Inoculated plants were placed in clear seedling trays under preinoculation conditions and monitored for aphid population changes every 24 h.

### *CsUGT89A2* gene suppression in tea leaves

AsODN targeting *CsUGT89A2* were designed using Soligo software (https://sfold.wadsworth.org/cgi-bin/soligo.pl;  Table S5) and synthesized by Tsingke Company (Beijing, China). To suppress *CsUGT89A2* expression in tea second leaves, 1 mL of 50 μM AsODN-*CsUGT89A2* solution was administered, with sODN used as controls. Each treatment was performed in triplicate. After 72 h, the samples were flash-frozen in liquid nitrogen and stored at −80°C for NBT staining and metabolomics analysis.

### ROS analyses

Nitroblue tetrazolium (NBT) staining: Immerse the tea plant leaves, as detailed in the preceding step, in a 50-mM sodium phosphate buffer with 0.05% (w/v) NBT for 24 h, followed by immersion in 75% ethanol at ambient temperature for 8 h. Subsequently, immerse the leaves in boiling water for 30 min until the stained regions exhibit a pronounced blue hue. Preserve the specimens in 75% ethanol and utilize a camera to document the stained regions.

### Chemical application on tea flowers and *T. aurantia* treatment

Fresh, unopened tea flowers (*Camellia sinensis* var. sinensis, cv. ‘Shuchazao’) were uniformly treated with a 0.03 mM flavonoid mixture (A, K, L and Q) or a glycoside mixture (A-7-G, K-7-G, L-7-G, and Q-7-G), both of which were diluted in sterile water. Treated flowers were incubated in 96-well plates with sterile water. After 48 h, samples were flash-frozen in liquid nitrogen and stored at −80°C for metabolic analysis. Thirty wingless adult *T. aurantia* aphids, starved for 2 h, were introduced onto the treated flowers. Feeding and reproduction behaviors were monitored at 24-h intervals to evaluate the effects of flavonoid aglycones and glycosides.

### *CsUGT89A2* silencing in tea flowers and *T. aurantia* treatment

To assess the impact of *CsUGT89A2* silencing on tea plant resistance to *T. aurantia*, the physiological responses of *T. aurantia* were assessed following gene suppression. Two specific AsODN primers targeting *CsUGT89A2* were designed based on its full-length sequence, with sODN serving as controls. Tea flowers were used as experimental material, and all primers were synthesized by Tsingke Biotech Co., Ltd. (Beijing, China). The flowers were independently treated with AsODN-*CsUGT89A2* or sODNs, sampled after 72 h, flash-frozen in liquid nitrogen, and stored at −80°C for transcript and metabolic analysis. Gene silencing efficiency was confirmed via qRT-PCR using the Bio-Rad CFX Connect system. Postsilencing, residual tea flower tissues were incubated at 25°C and 80% relative humidity for 48 h. Subsequently, *T. aurantia* individuals were introduced, and their behavioral activity was monitored and recorded at 24-h intervals.

### Quantitative RT-PCR analysis

Glyceraldehyde-3-phosphate dehydrogenase was used as the internal reference gene. qRT-PCR primers were designed using Primer 5.0, with primer sequences listed in Table S5. Total RNA was extracted from leaf and flower samples and reverse-transcribed into cDNA using PrimeScript. qRT-PCR was performed in triplicate on a StepOne Real-Time PCR system, and relative gene expression was calculated using the 2^−ΔΔCt^ method.

### Statistical analysis

All experimental data were evaluated utilizing Microsoft Excel (2024 version), with each experiment performed in triplicate or more. Statistical analysis was conducted utilizing Origin 2024b and SPSS 24.0, TBtools was utilized to quantify transcript quantity and form gene clusters, resulting in the generation of the heatmap. The post charts were generated using Adobe Photoshop 2021 and Adobe Illustrator 2021.

## Supplementary Material

Web_Material_uhaf212

## Data Availability

The data that support the findings of this manuscript are avail. The transcriptome data used in this article has been uploaded to the NCBI database and the CNCB database of the bioproject, with a total of 21 sets of raw data obtained, and the accession numbers are as follows *C. camelliae* I and II (PRJNA493214), *B. blight* I and II (PRJNA306068), *E. flavescens* (PRJNA553681), *E. grisescens* (CRA000859), *D. segeticola* (PRJNA528172), *G. blight* (PRJNA564655), Drought (PRJEB11522), IBA I and II (PRJNA240661), High-temperature drought (PRJNA545401), NAA (PRJNA690632), Salt (PRJNA387271), BRS (PRJNA756445), SA (PRJNA857833), Cold (GSE216311), Sugar (PRJNA381680), MeJA (PRJNA288922), Aluminum (PRJNA473596), Drought with FA (PRJNA596070). Additionally, in this study, the transcriptome data of the *T. aurantia* has been uploaded to the Sequence Read Archive database, with the accession number PRJNA1195731.
