# Supplementary material for: CsUGT89A2 enhances tea plant resistance to Toxoptera aurantia by mediating flavonoid glycosides biosynthesis
Source: Hortic Res. 2025 Aug 11;12(11):uhaf212. doi: 10.1093/hr/uhaf212 (PMC12577854; doi:10.1093/hr/uhaf212)

**Figure S1.** 12% SDS-PAGE electrophoretic analysis of recombinant CsUGT89A2 protein. M, protein molecular weight marker; lane 1, the supernatant of cultured cells not induced by IPTG; lane 2, the supernatant of cultured cells after induced by IPTG; lane 3, the precipitation of induced cells following sonication-driven cell disruption; lane 4, CsUGT89A2: purified recombinant rCsUGT89A2 protein approximately 53.6 kDa.

**
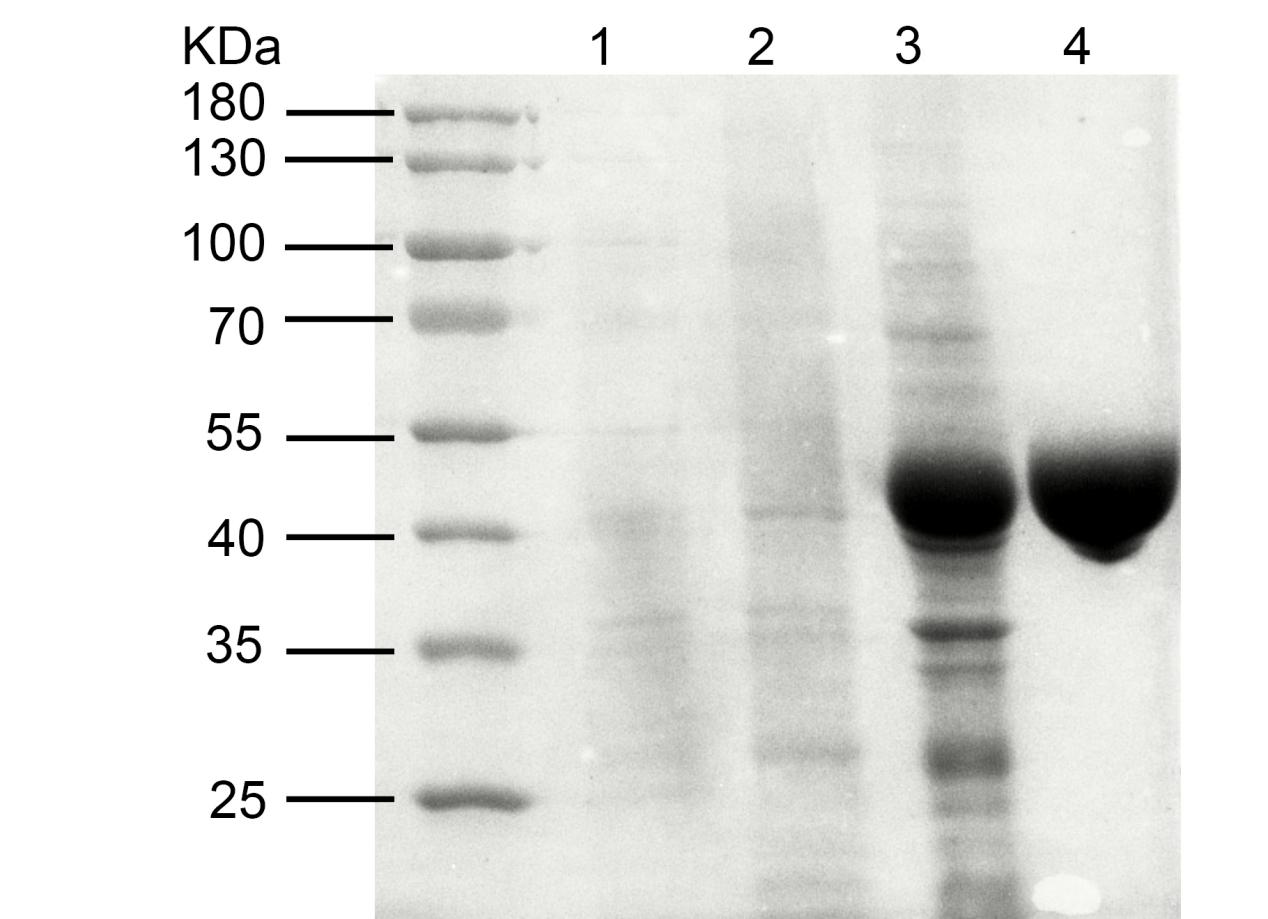
**

**Figure S2.** UPLC-Q-TOF-MS/MS analysis of the enzymatic product of rCsUGT89A2. (A) A-7-G: apigenin-7-*O*- glucoside. (B) K-7-G: kaempferol -7-*O*- glucoside. (C) L-7-G: luteolin-7-*O*-glucoside. (D) Q-7-G: quercetin-7-*O*- glucoside.


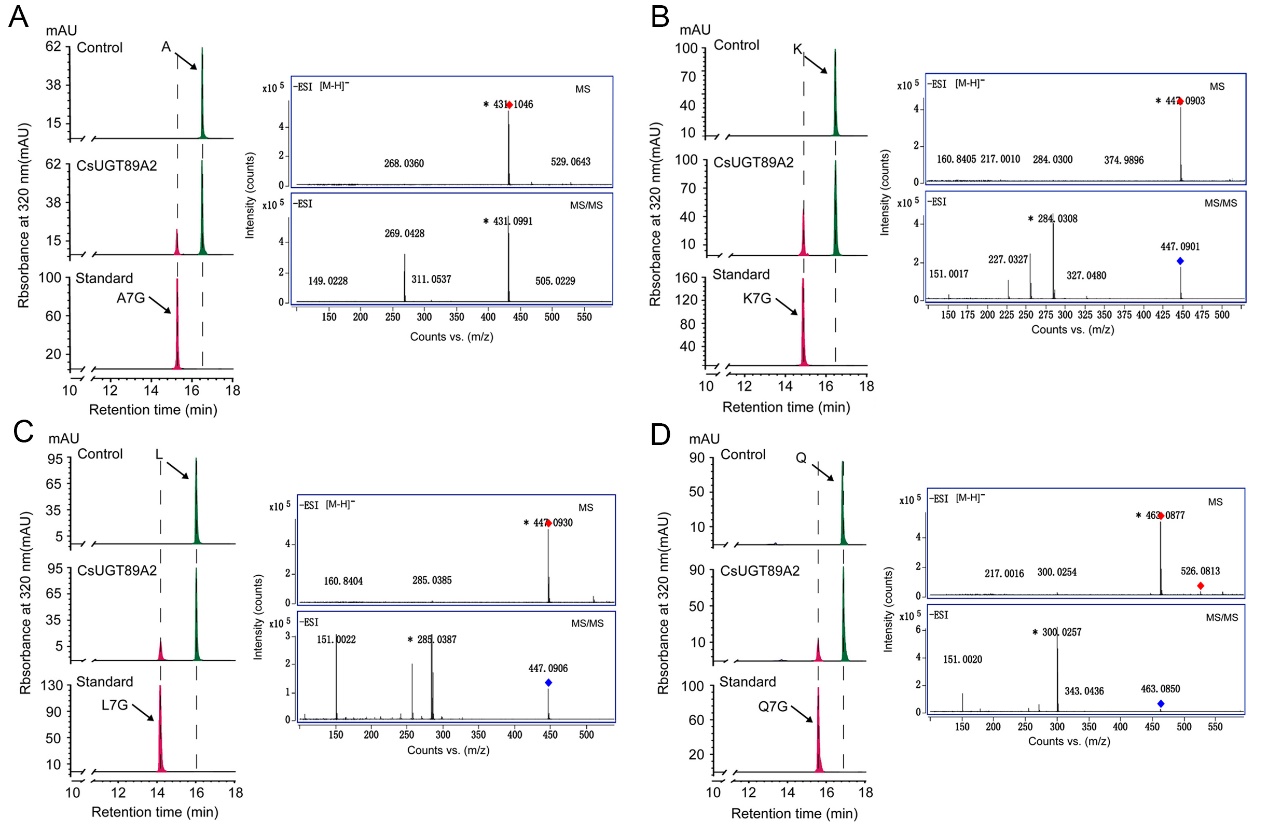

Supplement: Web_Material_uhaf212 [file web_material_uhaf212.zip › Supplementary Figure.docx]
